# Management of glaucoma with Boston type 1 keratoprosthesis

**DOI:** 10.5935/0004-2749.20230026

**Published:** 2023-04-10

**Authors:** Canan Asli Utine, Gül Arıkan, Elvan Yardım, Üzeyir Günenç

**Affiliations:** 1 Department of Ophthalmology, Dokuz Eylul University, Izmir, Turkey; 2 Izmir Biomedicine and Genome Center, Izmir, Turkey

**Keywords:** Glaucoma/surgery, Intraocular pressure, Postoperative complication, Prosthesis implantation, Glaucoma drainage implant, Glaucoma/cirurgia, Pressão intraocular, Complicação pós-operatória, Implantação de prótese, Implante para drenagem de glaucoma

## Abstract

**Purpose:**

To describe the frequency, clinical characteristics, complications, and management of glaucoma in eyes that underwent keratoprosthesis implantation.

**Methods:**

Patients who underwent keratoprosthesis surgery between June 2010 and January 2020 were retrospectively evaluated for glaucoma association and prognoses.

**Results:**

Among 17 patients who underwent keratoprosthesis surgery, 9 (52.9%) were associated with underlying or keratoprosthesis-induced glaucoma. Five eyes (29.4%) had underlying glaucoma and underwent a glaucoma drainage device implantation at least 6 months before keratoprosthesis surgery. One eye (5.9%) with normal intraocular pressure had glaucoma drainage device implantation at the same session with keratoprosthesis surgery due to high-risk characteristics of anterior segment structures. Four eyes with preexisting glaucoma showed progression after keratoprosthesis surgery. Additional antiglaucomatous treatment was commenced in two eyes whereas implantation of 2^nd^ glaucoma drainage device was performed in two eyes. Postoperative complications in three eyes (100%) with glaucoma drainage device implanted 6 months before or at the same session with aphakic type keratoprosthesis surgery with partial vitrectomy included rhegmatogenous retinal detachment in two eyes and bacterial endophthalmitis in one eye. Migration of silicone oil through the tube to the subconjunctival area was seen after pars plana vitrectomy in one eye. None of the three eyes (0%) that underwent glaucoma drainage device implantation years before keratoprosthesis surgery experienced a posterior segment complication other than glaucomatous progression. Out of 11 eyes with no previous history of glaucoma, 3 (27.3%) showed high intraocular pressure and glaucomatous disc changes after keratoprosthesis surgery, which could be pharmacologically controlled.

**Conclusions:**

In this cohort, eyes with preexisting glaucoma were more difficult to manage compared to eyes with *de novo* glaucoma after keratoprosthesis surgery. Retinal complications appeared more often when glaucoma drainage device implantation was performed no more than 6 months before aphakic type keratoprosthesis surgery with partial vitrectomy.

## Abstract (pt)

**Objetivo:**

Descrever a frequência, as características clínicas, as complicações e o manejo do glaucoma em olhos submetidos a implantes de ceratoprótese.

**Métodos:**

Pacientes submetidos à cirurgia de ceratoprótese entre junho de 2010 e janeiro de 2020 foram avaliados retrospectivamente em termos de glaucoma associado e prognóstico.

**Resultados:**

Dos 17 pacientes submetidos à cirurgia de ceratoprótese, em 9 (52,9%) foi constatado glaucoma subjacente ou induzido por ceratoprótese. Cinco olhos (29,4%) tinham glaucoma subjacente e receberam a implantação de um dispositivo de drenagem de glaucoma pelo menos 6 meses antes da cirurgia de ceratoprótese. Um olho (5,9%) com pressão intraocular normal teve implantado um dispositivo de drenagem de glaucoma na mesma sessão da cirurgia de ceratoprótese, devido às características de “alto risco” das estruturas do segmento anterior. Quatro dos olhos com glaucoma preexistente apresentaram progressão após a cirurgia de ceratoprótese. Foi iniciado um tratamento antiglaucomatoso adicional em 2 olhos, enquanto outros 2 olhos receberam o implante de um segundo dispositivo de drenagem de glaucoma. Foram observadas complicações pós-operatórias em 3 olhos (100%) com dispositivo de drenagem de glaucoma implantado 6 meses antes ou na mesma sessão da cirurgia de ceratoprótese tipo afácica com vitrectomia parcial, incluindo descolamento de retina regmatogênico em 2 olhos e endoftalmite bacteriana em 1 olho. Em 1 olho observou-se migração do óleo de silicone para a área subconjuntival através do tubo após vitrectomia via *pars plana*. Nenhum dos 3 olhos (0%) implantados com dispositivo de drenagem de glaucoma anos antes da cirurgia de ceratoprótese apresentou complicações do segmento posterior, exceto progressão glaucomatosa. Dos 11 olhos sem história prévia de glaucoma, 3 (27,3%) apresentaram alta pressão intraocular e alterações do disco glaucomatoso após cirurgia de ceratoprótese, condições que podem ser controladas clinicamente.

**Conclusões:**

Nesta coorte, os olhos com glaucoma pré-existente foram mais difíceis de manejar, comparados àqueles que desenvolveram glaucoma após a cirurgia de ceratoprótese. Apareceram mais complicações retinianas quando o implante do dispositivo de drenagem de glaucoma foi realizado no máximo 6 meses antes da cirurgia de ceratoprótese do tipo afácico com vitrectomia parcial.

## INTRODUCTION

Glaucoma is one of the most common reasons for irreversible vision loss after Boston keratoprosthesis (Kpro) surgery^(1^, ^2^, ^3^, ^4)^. Among patients referred to Kpro surgery, up to 3/4^th^ already have glaucoma^(4^, ^5^, ^6^, ^7^, ^8)^ while on average, 1/4^th^ of cases develop *de novo* glaucoma after Kpro implantation^(6)^. High prevalence, rapid progression, difficulty in intraocular pressure (IOP) assessment, and lack of standardized treatment algorithm make this potentially blinding problem a challenge. Even in eyes that were under control preoperatively, glaucoma tends to show progression postoperatively. Furthermore, eyes with *de novo* glaucoma after Kpro are known to show similar rates of progression as those with preexisting glaucoma^(2)^. Therefore, a low threshold for glaucoma drainage device (GDD) implantation has been recommended before or concomitantly with Kpro surgery^(2^, ^3^, ^4)^. Indeed, all Kpro eyes should be considered at high risk for glaucoma and followed up with periodic IOP measurement, visual field (VF) analysis, and optic disc imaging^(4)^.

This study aims to investigate the frequency of Kpro glaucoma association, modes of management, and associated complications in our cohort of Boston type 1 Kpro patients. Particularly, we comparatively evaluated the prognosis of eyes with preexisting and post-Kpro glaucoma, and prognosis with respect to the timing of GDD surgery.

## METHODS

This study included retrospective evaluation of patients who underwent Boston type 1 Kpro surgery between June 2010 and January 2020. Patients’ demographic properties, including indications for Kpro implantation, modes of their glaucoma management, and visual and anatomic prognoses, were evaluated. The study adhered to the Tenets of Helsinki.

Patients who were deemed inoperable with corneal allograft surgeries were evaluated for a possible Kpro surgery. Kpro-candidate patients were informed that future surgeries may be required for any Kpro-associated complication, including glaucoma. Signed informed consent was obtained preoperatively.

Kpro surgeries were performed as described in the literature^(9)^, by the same surgeon (CAU). All patients received a titanium backplate of 8.5 mm diameter and threadless-design Boston type 1 Kpro implantation. Patients with concomitant cataracts underwent combined extracapsular cataract extraction and aphakic type Kpro implantation surgeries. Previously implanted intraocular lens (IOL) was removed if it was decentralized, followed by an aphakic type Kpro surgery. In-the-bag and centralized IOLs were kept in place with pseudophakic Kpro surgery. In eyes with severe desiccating dryness (i.e., signs of reduced tear volume and tear turnover rate, increased corneal surface irregularity, disruption of corneal epithelial barrier function, and conjunctival squamous metaplasia), a modified Kpro surgery was performed, where the ocular surface was totally covered with a conjunctival Gunderson flap^(10)^ or buccal mucosa if there was inadequate healthy conjunctiva. Central trephination over Kpro optic was performed 3 months postoperatively after vascularization took place.

Postoperatively, all eyes were maintained on topical moxifloxacin, vancomycin, and prednisolone acetate 4 times daily, and a contact lens over the Kpro, except for those with a modified Kpro. Patients underwent complete ophthalmological examination at each follow-up visit that included best-corrected visual acuity (BCVA) assessment in Snellen lines, anterior segment slit-lamp examination, fundus examination with indirect ophthalmoscopy, cup to disc (c/d) ratio assessment, VF examination (Humphrey Field Analyzer 3, Zeiss), swept-source optical coherence tomography (OCT) imaging of the macula and optic disc with retinal nerve fiber layer (RNFL), and ganglion cell complex analyses (DRI OCT Triton, Topcon).

Preoperative IOP was measured with Goldmann applanation tonometer or TonoPen depending on the severity of the corneal disease. After Kpro surgery, IOP was assessed by digital palpation and TonoPen measurements at the temporal sclera. In the digital palpation method, IOP was defined as low if estimated <10 mmHg, normal if estimated as 10-20 mmHg, and high if estimated >20 mmHg. A constant value of 15 mmHg was subtracted from all measurements obtained with TonoPen through the sclera and recorded as the IOP level.

**Figure 1:**
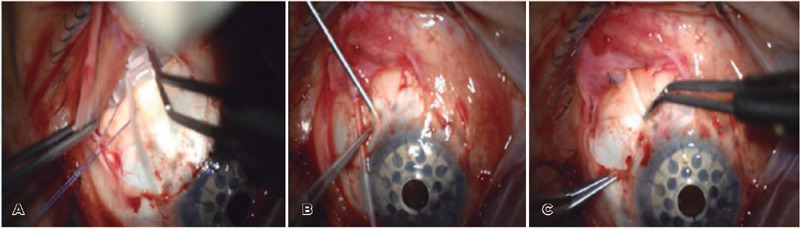
A-C) Ahmed Glaucoma Valve implantation steps in an eye with preexisting Boston type 1 Keratoprosthesis (patient 3).

Modes of management in recalcitrant cases included implantation of a GDD (Ahmed® Glaucoma Valve, New World Medical, Inc., CA, USA) before, concurrently, or after Kpro surgery (Figure 1 A-C) or transscleral cyclophotocoagulation (CPC) after Kpro surgery.

The data were collected retrospectively from the patient records and tabulated in an Excel sheet using Microsoft Excel 2016. All Kpro eyes within the study period were retrospectively reviewed since each of them had records of careful preoperative and postoperative follow-up visits for at least one year. Patients who had preexisting glaucoma and those who developed glaucoma after Kpro surgery were included in this study. Those patients who showed neither preoperative nor postoperative glaucomatous signs were excluded from the analysis.

In this descriptive study, values were expressed as median, range, and frequency rates. No further statistical analysis was performed due to a small number of included cases.

## RESULTS

A total of 17 monocular patients who underwent Kpro surgery were followed up for a median of 3 years (range: 1-10 years). Etiologies included a history of recurrent immunologic graft rejection in seven eyes, autoimmune ocular surface disease in four eyes, severe limbal stem cell deficiency secondary to alkaline burn in three eyes, thermal burn in one eye, intense topical antiglaucomatous use in one eye, and prephthisis with aphakia and intraocular silicone oil in one eye.

Demographic properties of nine Kpro patients (9/17, 52.9%) who were associated with glaucoma are shown in Table 1. Postoperatively, median best-ever BCVA was 0.6 (range: 0.3-1.0), and final BCVA was 0.4 (range: light perception to 0.7). At the last follow-up, 88.89% of eyes (8/9) had improved vision compared to preoperatively.

**Table 1:** Demographic features and examination findings of Kpro patients with glaucoma

Patient #	Gender	Age	Etiology	Pre-Kpro corneal graft #	Follow-up after Kpro surgery	Initial BCVA	Final BCVA	Best-ever BCVA	Final exam notes
1	F	41	KCN, multiple graft failure, decentralized PCIOL	4	3 years	10 cmcf	0.5	1.0	Kpro intact, aphakic, subconjunctival SO, retina flat after PPV for RRD and SO removal, thin ERM, c/d 0.4, 2 GDDs
2	M	59	Bullous keratopathy, multiple graft failure, decentralized PCIOL Multiple graft failure,aphakic	2	4.5 years	HM	LP	0.6	Kpro intact, aphakic, closed tunnel retinal detachment due to endophthalmitis Kpro intact, aphakic,c/d 0.9, 2 GDDs
3	F	42	Multiple graft failure,aphakic	3	3 years	HM	0.5	0.5	Kpro intact, aphakic,c/d 0.9, 2 GDDs
4	M	78	Trachoma, multiple graft failure, decentralized PCIOL	2	3 years	LP	0.05	0.3	Kpro intact, aphakic, retina flat after PPV for RRD, c/d 0.3
5	M	64	KCN, multiple graft failure, PCIOL	2	3.5 years	1 mcf	0.4	0.6	Kpro intact, pseudophakic, c/d 0.7 Severely constricted VF
6	M	69	Severe LSCD due to topical antiglaucomatious drugs use, PCIOL	0	2.5 years	HM	0.1	0.3	Kpro intact, pseudophakic, c/d 0.9–1.0
7	M	47	Chemical burn, graft failure, decentralized PCIOL	2	3.5 years	HM	0.7	0.7	Kpro intact, aphakic, c/d 0.9,Severely constricted VF
8	M	47	VKC, KCN, multiple graft failure, PCIOL	3	3 years	HM	0.7	1.0	Kpro intact, pseudophakic, c/d 0.4, Severely constricted VF
9	F	51	Severe AKC, multiple graft failure, nuclear cataract	4	1 year	10 cmcf	0.1	0.4	Kpro intact, aphakic, c/d 0.5

Kpro= keratoprosthesis; BCVA= best-corrected visual acuity; F= female; KCN= keratoconus; PCIOL= posterior chamber intraocular lens; cmcf= centimeters counting fingers; SO= silicone oil; PPV= pars plana vitrectomy; RRD= rhegmatogenous retinal detachment; ERM= epiretinal membrane, c/d= cup to disc ratio, GDD: glaucoma drainage device; M= male; HM= hand motion; LP= light perception; mcf: meters counting fingers; VF= visual field; LSCD= limbal stem cell deficiency; VKC= vernal keratoconjunctivitis; AKC= atopic keratoconjunctivitis. * Pre-GDD IOPs after Kpro implantation and post-GDD IOPs were measured through the temporal sclera by TonoPen® as described in the text.

### Kpro patients with underlying or at high risk for glaucoma

Three out of four patients (75%) who required a modified Kpro surgery due to severe dry eye and ocular surface disease (patients 4, 7, and 9) required either a pre-Kpro or simultaneous GDD implantation surgery or postoperative pharmacological treatment for glaucoma, respectively.

A total of five eyes (5/17, 29.4%) had underlying glaucoma, and underwent implantation with a GDD at least 6 months before Kpro surgery. Only one eye (1/17, 5.9%) had GDD implantation at the same session with Kpro surgery, although IOP was normal preoperatively, due to high-risk characteristics of his anterior segment structures with extensive peripheral anterior synechiae. The tube was placed into the posterior chamber through the ciliary sulcus (Table 2).

**Table 2:** Demographic features and examination findings of patients with glaucoma or were at high risk for glaucoma before Kpro surgery

Patient #	History of glaucoma before Kpro	Time for GDD implantation with respect to Kpro	Pre-Kpro IOP (mmHg)	Pre-GDD IOP* (mmHg)	Post-GDD final IOP* (mmHg)	Final antiglaucomatous medication	Other comments
1	Yes	Six months before and three years after	18	20	12	None	Postoperative RRD, treated with PPV. Silicone oil occluded the GDD. 2^nd^ GDD implanted
2	Yes	Six months before	12	28	10	None	No further IOP elevation
3	Yes	1.5 year before and six months after	15	24	12	dorzolamide 2% + timolol 0.5% brimonidine 0.15 % travoprost 0.004 %	A session of transscleral CPC performed for IOP elevation; but no permanent effect. 2^nd^ GDD implanted.
4	No	At the same session with Kpro implantation	20	20	9	None	Postoperative RRD, treated with PPV. Remained hypotonic thereafter.
6	Yes	Two years before	14	Unknown	22	brinzolamide 1% + timolol 0.5% brimonidine 0.15 % travoprost 0.004 %	Additional topical antiglaucomatous treatment for IOP control
7	Yes	Seven years before	12	Unknown	15	brinzolamide 1% + timolol 0.5 %acetazolamide 250 mg (qd)	GDD surgery was recommended but denied due to “high risk” characteristics of ocular surface disease

Kpro= keratoprosthesis; GDD= glaucoma drainage device; IOP= intraocular pressure; RRD= rhegmatogenous retinal detachment; PPV= pars plana vitrectomy; CPC= cyclophotocoagulation; qd= one per day* Pre-GDD IOPs after Kpro implantation and post-GDD IOPs were measured through the temporal sclera by TonoPen® as described in the text.

During postoperative visits, two eyes (2/6, 33.3%) did not experience further IOP elevation, but in two eyes (2/6, 33.3%), additional antiglaucomatous treatment was commenced. Glaucoma surgery was recommended in three eyes (3/6, 50%), but one patient denied further surgery. Second GDD implantation at the inferotemporal quadrant was performed in two eyes (2/6, 33.3%), one of which was after an inadequate transscleral CPC.

Two eyes (2/6, 33.3%) presented with rhegmatogenous retinal detachment (RD) 16 months after Kpro surgery, and underwent successful vitreoretinal surgeries. In patient 1, a GDD had been implanted 6 months before Kpro surgery. Following vitreoretinal surgery, silicone oil migration through the tube shunt into the bleb and subconjunctival area was noted 5 months after vitrectomy resulting in IOP elevation and poor fit of the contact lens due to conjunctival elevation. Subconjunctival cleaning did not result in IOP reduction and the 2^nd^ GDD implantation was performed. The only eye (patient 4) that had combined GDD and Kpro surgeries also developed RD and underwent vitreoretinal surgery without intraocular silicone oil injection, and remained with a low IOP during the follow-up.

One eye (1/6, 16.67%, patient 2) had endophthalmitis due to *S. aureus* infection and associated RD at 31 months postoperatively. He had quitted vancomycin and switched to aminoglycoside prophylaxis on his own will despite warnings two months before. His final vision was at the level of light perception in this eye, which retained a low IOP level.

### Eyes that developed glaucoma after Kpro surgery

Among 11 eyes without previous history of or high risk characteristics for glaucoma, three eyes (3/11, 27.3%) showed high IOP and glaucomatous disc changes after Kpro surgery, which could be controlled with topical antiglaucomatous agents in two eyes (66.7%) but required oral and intravenous medication in one eye (33.3%) to halt glaucoma attacks. None of the eyes that developed post-Kpro glaucoma necessitated surgery (Table 3).

**Table 3:** Demographic features and examination findings of patients who developed glaucoma after Kpro surgery

Patient #	History of glaucoma before Kpro	Time for GDD implantation with respect to Kpro	Follow-up after Kpro surgery	Pre-Kpro IOP (mmHg)	Final IOP * (mmHg)	Final antiglaucomatous medication
5	No	None	3.5 years	17	11	dorzolamide 2% + timolol 0.5%brimonidine 0.15% latanaprost 0.005%
8	No	None	3 years	12	14	dorzolamide 2% + timolol 0.5%brimonidine 0.15%
9	No	None	1 year	12	17	brinzolamide 1% + timolol 0.5%brimonidine 0.15% High IOP attacks were managed with I.V. mannitol and P.O. acetazolamide

Kpro= keratoprosthesis; GDD= glaucoma drainage device; IOP= intraocular pressure; I.V.= intravenous; P.O.= per oral.

* Final IOPs were measured through the temporal sclera by TonoPen® as described in the text.

## DISCUSSION

Glaucoma is currently one of the biggest challenges in preserving visual improvement after Kpro surgery. The rate of c/d ratio progression in glaucomatous Kpro eyes was reported to be approximately 7 times faster compared to that in patients with primary open-angle glaucoma, the fastest in Kpro patients secondary to corneal burns due to postinjury release of inflammatory mediators that may cause direct damage to and/or apoptosis of retinal ganglion cells, and the slowest in those without a history of prior corneal surgery^(6,11)^. Mechanism of *de novo* glaucoma after Kpro surgery is multifactorial, with iridocorneal angle damage^(6)^ being the most emphasized. Iridocorneal adhesions^(12)^, vitreous or inflammatory debris obstructing trabecular meshwork^(6,7)^, chronic angle closure due to ongoing inflammation^(4)^, particularly in patients with a history of failed penetrating grafts^(13)^, anterior segment crowding by a large Kpro backplate impeding aqueous outflow^(7)^, collapse of trabecular meshwork scaffold in aphakic eyes^(14)^, angle closure by a residual iris stump^(13)^, as well as) and chronic use of topical steroids before Kpro surgery^(13)^ have been implicated in the development of *de novo* glaucoma after Kpro surgery. Kpro implant-induced alternation of scleral rigidity may also cause biomechanical damage at the level of lamina cribrosa^(7)^ and lead to progressive optic neuropathy despite adequate IOP control. In this cohort, 3 of 4 eyes (75%) that required a modified Kpro surgery were associated with glaucoma, owing to their aggressive ocular surface and anterior segment conditions, requiring appropriate management.

The main obstacle in glaucoma assessment is the inability to directly measure IOP through Kpro optic. Estimation by digital palpation is the most practical method for assessment of IOP in clinical practice, but it is generally useful only for detection of markedly elevated IOP over 30 mmHg^(4)^. In this method, one should avoid digital palpation over Kpro optic, backplate, or near-GDD plate as GDD placement may alter scleral dynamics. On the other hand, pneumotonometer and TonoPen use on the sclera in Kpro patients tend to overestimate IOP^(4)^ similarly to the 9 mmHg higher scleral pneumotonometry results in comparison with corneal pneumotonometry that shows a linear correlation in treatment-naïve eyes^(15)^. In eyes with Boston type 1 Kpro, Schiotz tonometry on the temporal sclera or corneoscleral limbus, was shown to have higher accuracy than TonoPen, in comparison with the ‘gold standard digital manometry^(16)^. With any technique of limbal or scleral measurement, comparison with the same location measurements from the fellow eye is recommended for accurate interpretation of readings. Integration of a fiber-optic pressure sensor for real-time IOP measurement^(17)^ might be of help in the future once retroprosthetic membrane formation can be controlled.

Serial optic disc photos and OCT analysis of RNFL thickness and volume, c/d ratio, and cup volume data and macular ganglion cell complex analysis are useful tools for evaluation of disease progression when clear optic of the Kpro implant allows imaging^(18)^. Anterior segment OCT and ultrasonic biometry can be employed to evaluate angle structures and GDD positioning^(19)^ whereas Goldmann VF would allow functional assessment^(20)^. In our cohort, 3 patients had severely constricted VF despite BCVA ≥0.6 and good IOP control (Table 1).

Topical antiglaucomatous medications are less effective in eyes with a Kpro due to the reduced ocular surface available for eye drop absorption. Systemic carbonic anhydrase inhibitors were proposed when additional pharmacological therapy is necessary^(4)^. Two of our cases (patients 7 and 9) have been long under oral acetazolamide treatment without side effects.

Debate is going on about the type and timing of glaucoma surgery^(6,21)^. More than 10% of patients were reported to require glaucoma surgery following Kpro surgery^(4,5,21)^ with particular success with valved GDD placement^(13,22)^, as trabeculectomies are less effective because of tissue scarring^(4)^. High incidence and severity of glaucoma have prompted a low threshold for performing surgery either before or simultaneously with Kpro^(13)^. Studies suggest lower IOP, slower progression in c/d ratio^(6,23,24)^, and better preservation of vision^(25)^ with aggressive perioperative management of glaucoma. Even one study recommended prophylactic vitrectomy and GDD placement prior to Kpro in eyes without glaucoma^(26)^.

Combined GDD and Kpro surgery provides an opportunity for additional IOP management and restoring vision at the same session^(24)^. However, studies also report positive correlation of GDD-related complications and vision loss with combined surgery; the most frequent of which is GDD erosion, in ~1/4^th^ of patients in one study^(25)^. Postoperative mandatory use of a bandage contact lens increases this risk, likely as a result of mechanical contact^(8)^. A fibrous capsule surrounding GDD might form in eyes with cicatrizing conjunctival disease and decrease the effectiveness of the tube^(4)^. Therefore, in eyes without a history of glaucoma before Kpro, GDD implantation could be deferred until definitive evidence of glaucomatous damage develops. Nevertheless, glaucoma surgery in a Kpro eye can be more challenging when the viewing area is limited to 3 mm. Ensuring an adequate shave of vitreous base for pars plana placement and correct location of the occluded tube for Nd:YAG laser^(27)^ may be challenging.

Tube occlusion was reported to be the most common complication of all GDDs in Kpro eyes^(4)^. If GDD is placed in the anterior chamber, posterior chamber IOL will protect against tube occlusion from anterior migration of the vitreous, and a peripheral iridectomy will protect against occlusion by intact iris. Sulcus placement has been recommended to prevent anterior chamber crowding with a long tube in radial orientation so that the tip is visible with reduced contact with Kpro backplate^(13)^, as in patient 4 with extensive peripheral anterior synechia. A core vitrectomy has been considered adequate in aphakic eyes, although pars plana GDD should be accompanied by a complete vitrectomy to decrease the high risk of vitreous incarceration^(8,28)^.

Notably, in this cohort, all three eyes (100%) that had GDD implantation surgery 6 months before or simultaneously with Kpro surgery had RD. One of them (patient 2) was associated with bacterial endophthalmitis. Two eyes (patients 1 and 4) with aphakic type Kpro after explantation of their decentralized IOLs and meticulous anterior vitrectomy had rhegmatogenous RD. One may argue that if the eye is to be left aphakic, a complete pars plana vitrectomy rather than core vitrectomy will secure the patency of the tube. On the other hand, none of the three eyes (0%) that received GDD implantation years before Kpro surgery experienced a posterior segment complication other than glaucomatous progression. Two of these eyes (patients 3 and 7) underwent IOL explantation with anterior vitrectomy and aphakic Kpro implantation whereas in one eye (patient 6), a stabile posterior chamber IOL was preserved. Intraocular fluid dynamics may have already stabilized at equilibrium in these eyes and did not create traction on the retina. It should also be noted that in none of the 6 eyes that had GDD surgery, a complete vitrectomy was performed. GDD and aphakic Kpro surgeries within 6 months with only partial vitrectomy may create unsafe intraocular fluidics and lead to posterior segment complications.

In this cohort, the frequency of *de novo* high IOP development that required topical and oral antiglaucomatous agents was 27.3% (3/11). In two eyes with multiple graft rejections, IOP was pharmacologically controlled. In one eye with severe cicatrizing keratoconjunctivitis (patient 9), glaucoma was successfully managed with topical and systemic agents. GDD implantation or transscleral CPC was deferred not to disturb conjunctival homeostasis.

Transscleral CPC was reported to normalize IOP in 66.67% of eyes with Kpro-associated glaucoma^(21)^. It is a good therapeutic option for type II Kpro in which the conjunctiva has been resected^(29)^ or in cases of type I Kpro if the conjunctiva is sufficiently scarred^(4)^. Endoscopic CPC was even suggested as the first-line treatment in Kpro eyes^(7)^ with the benefit of no permanent hardware. However, difficult titration, requiring multiple procedures^(13)^, and excessive ciliary body damage that can result in hypotony are the disadvantages. Caution must be taken for microbial infection due to lack of biointegration^(30)^. Here, only patient 3 with pre-Kpro GDD underwent a session of transscleral CPC after Kpro. However, the effect was not long-lasting as implantation of the 2^nd^ GDD in addition to medications was needed.

We acknowledge the limitations of our study, such as a small number of cases that render it impossible to perform statistical analysis. Patients also had relatively short follow-ups. On the other hand, our cohort included a variety of underlying etiologies, and enabled us to describe the frequency of glaucoma, possible modes of management, and complications.

In conclusion, in this cohort, further glaucoma surgeries were required in half of the cases with preexisting glaucoma and pre-Kpro GDD unlike in post-Kpro glaucoma cases. Retinal complications appeared more when GDD implantation was performed no more than 6 months before aphakic type Kpro surgery with partial vitrectomy.
